# COVID-19-Related distress, body image, and eating behaviors: a cross-sectional explanatory model

**DOI:** 10.1186/s40359-024-01613-z

**Published:** 2024-03-02

**Authors:** Christopher Rodrigue, Rachel F. Rodgers, Noémie Carbonneau, Catherine Bégin, Jacinthe Dion

**Affiliations:** 1 Department of Psychology, University of Quebec at Trois-Rivières, 3351 des Forges Blvd, Trois-Rivières, QC G8Z 4M3 Canada; 2 Faculty of Agriculture and Food Sciences, Laval University, 2440 Hochelaga Blvd, Québec, QC G1V 0A6 Canada; 3 Department of Applied Psychology, Northeastern University, 360 Huntington Avenue, MA02115, Boston, MA USA; 4 Intersectional Center for Sustainable Health, University of Quebec at Chicoutimi, 555 de l’Université Blvd, Chicoutimi, QC G7H 2B1 Canada; 5 Interdisciplinary Research Center on Intimate Relationship Problems and Sexual Abuse, Department of Psychology, University of Montreal, 90 Vincent d’Indy, Montreal, QC H2V 2S9 Canada

**Keywords:** COVID-19, Body image, Eating behaviors, Social media, Mindfulness, Adolescents

## Abstract

**Background:**

The COVID-19 pandemic has been associated with increased body dissatisfaction and disordered eating behaviors in adolescents. To better understand these associations, this study tested an explanatory model in which appearance-focused social media use, internalization of social media pressure, and mindfulness abilities mediated the relationship between COVID-related distress and body dissatisfaction, which in turn was associated with dietary restraint and binge eating episodes.

**Methods:**

Adolescents (*N* = 493, *M*_age_= 16.7; 47.5% girls) recruited within high schools completed online measures. We conducted model testing through path analysis in MPlus, using modification indices to derive a well-fitting model.

**Results:**

The initially hypothesized model was a poor fit for the data. The final well-adjusted model confirmed several significant paths and supported the parallel mediational roles of social media (specifically, the use of appearance-focused social media and internalization of social media pressure) along with mindfulness, on the relationship between COVID-19-related distress and body satisfaction. Model adjustments involved adding three paths, resulting in two additional significant indirect effects, and suppressing one path.

**Conclusions:**

Findings suggest that mindfulness, media use and the internalization of social media pressure are potential key processes explaining body dissatisfaction and eating disorders among adolescents who experienced higher levels of COVID-related distress.

**Supplementary Information:**

The online version contains supplementary material available at 10.1186/s40359-024-01613-z.

## Introduction

Since December 2019, the coronavirus disease 2019 (COVID-19) pandemic has changed the way people live their lives, worldwide. In Canada, in response to this major health crisis, governments implemented several restrictive measures to reduce social contacts and control the spread of the virus. These consisted of intermittent periods of lockdown (partial to total) for many weeks, during which almost everything was closed (e.g., schools, malls, theaters, social, recreational, and cultural centers, restaurants, and bars). Since then, around the world research attention has been paid, not only on the impacts of the virus itself on the population, but also the collateral effects of these public health restrictions [1–3]. Many studies highlighted the impacts on adolescents in terms of physical, psychological, and social health [4–6]. Indeed, this major developmental stage is characterized by changes on multiple levels (biological, social, and affective), including the presence of more stressors (e.g., puberty and body changes, school requirements, part-time jobs, relationships, autonomy seeking) [7, 8]. The ongoing stress and uncertainty related to the COVID-19 pandemic and the social restrictions have undoubtedly added burden to this already challenging stage of life. The present study aimed to deepen our knowledge about the psychological impacts of this pandemic on adolescents, who are going through an important and stressful period of their development.

In terms of behavioral and mental health, since the COVID-19 outbreak, adolescents have reported decreased physical activity [9], isolation, depressive and anxiety symptoms [10–12], and have engaged in more screen time [13–15]. Some studies have also documented an increase in disordered eating behaviors and body image disturbances [2, 16, 17]. A recent scoping review concluded that the associations between the COVID-19 pandemic and increased in disordered eating behaviors (i.e., dietary restraint, compensatory behaviors, overeating) and body image concerns was strongest in vulnerable groups, such as adolescents with a history or an actual eating disorder or existing disordered eating, and adolescents with higher levels of body dissatisfaction and/or COVID-19-related anxiety/fear [18]. Furthermore, aspects of the pandemic most often associated with those changes were the following: changes in routine and lack of a normal structure (e.g., school, leisure time, physical activity), stressful environment, and difficulties in emotion regulation [19, 20]. However, the mechanisms that could explain how the COVID-19 pandemic is associated with eating behaviors and body image are still unclear.

Rodgers, Lombardo and their colleagues [21] proposed theoretical pathways through which the pandemic may have led to more image disturbances and disordered eating; one of them included social media exposure as a potential explanatory factor. Many studies conducted even before the COVID-19 pandemic, revealed that greater social media use was associated with body dissatisfaction and disordered eating in adolescents [22–25]. This body of literature led to the conceptualization of a biopsychosocial model linking, among other variables, social media use, body image concerns and body change behaviors (i.e., dietary restraint and muscle building behaviors) [26]. The model highlighted the predictive value of negative affect and social media use on body change behaviors, and the potential mediational role of social media pressure internalization and body dissatisfaction on these relationships. Recent research has examined the relationships between social media use and body image concerns in adolescents in context of the COVID-19 pandemic. For example, a study involving a large sample of Spanish young women (14–35 years), has observed an increase in the frequency of use of various social network sites during the lockdown [27]. Furthermore, for younger women (14–24 years old), the frequency of Instagram use was related to body dissatisfaction, drive for thinness and low self-esteem. More recently, Schmid and colleagues [28] reported that 40% of their U.S. sample of adolescents and young adults adopted weight and shape control behaviors during the pandemic, and almost half of the participants reported seeing posts about weight and/or shape on social media. The authors conclude that social media use might thus be a factor contributing to body image concerns in adolescents. In connection with these results, there is growing evidence suggesting that the type of social media content consumed is closely linked with body image and eating behaviors. For instance, a recent study by Sanzari et al. (2023) concluded that increased consumption of weight loss-related content was significantly linked to decreased body satisfaction and more frequent binge eating episodes, which was not related to time spent on social media [29]. A recent scoping review conducted by Dane & Bathia (2023) revealed similar findings, suggesting that social media content associated with body-control trends (such as “fitspiration” and “thinspiration”) and the use of appearance-focused social media were linked to heightened body image concerns and disordered eating [30]. They also identified internalization of social media pressure as a potential mediator in this relationship. Given the available data on the impacts of the COVID-19 pandemic on adolescents’ body image and eating behaviors, along with the role of social media (including both its use and the internalization of social media pressure) therein, it would be useful to unpack these relationships and clarify the underlying mechanisms.

Up to now, the COVID-19 pandemic has caused negative affects for many people [31, 32]. Among adolescents, research has suggested associations between emotional distress and both body image concerns and disordered eating during the pandemic, as well as more pronounced difficulties in emotion regulation [18]. Emotion regulation is a complex construct, defined as the capacity to manage emotions and control impulsive behaviors when experiencing negative feelings; it includes many facets, such as awareness and identification, understanding and acceptance of an emotional experience [33]. On the other hand, mindfulness is the ability to pay attention to internal and external states and emotions in the present moment, without judgment and reaction [34]. Studies have shown that mindfulness is strongly associated with emotion regulation and psychological health and is widely considered as an adaptive emotion regulation strategy [35]; it has been associated with fewer psychological difficulties, including eating disorders [36]. Furthermore, growing evidence has pointed to mindfulness as an effective intervention target to improve emotion regulation and psychological health [37–42]. A recent study showed positive effects of mindfulness-based interventions on COVID-19-related distress and global mental health during this period [43]. Thus, mindfulness represents an interesting avenue to understand the processes underlying the relationships between COVID-19-related distress, body dissatisfaction and disordered eating.

**Fig. 1:**
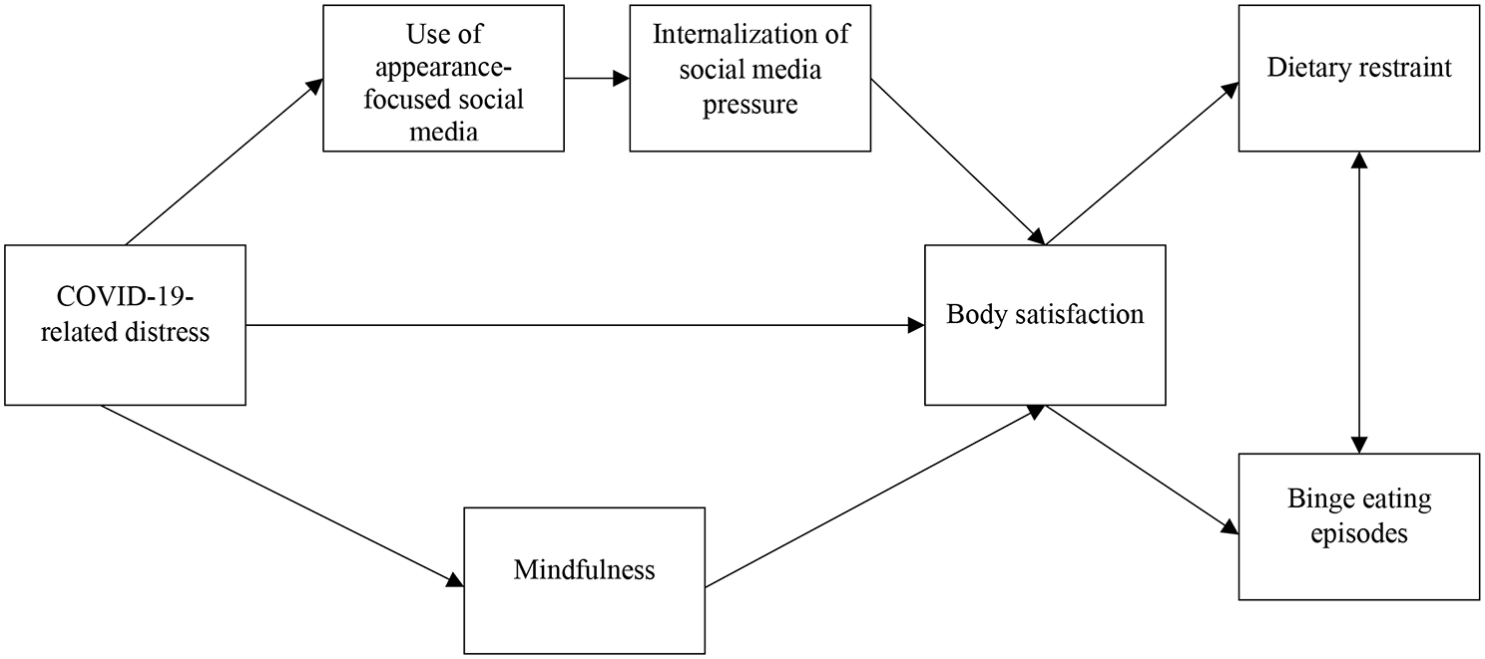
Initial model

The general aim of the present study was to examine the relationships between COVID-19 pandemic-related distress, social media use, mindfulness abilities, body image and disordered eating behaviors in a sample of Canadian adolescents from the general population. Specifically, we tested an explanatory model in which COVID-19-related distress was associated with body satisfaction, which in turn was associated with disordered eating (dietary restraint and binge eating episodes). To provide a deeper understanding of those relationships and identify potential underlying processes, the indirect effect of social media (i.e., social media use, internalization of social media pressure according to shape and weight), and of mindfulness were included in the model (see Fig. 1). Specifically, our hypothesis initially proposed a significant serial mediation effect involving the use of appearance-focused social media and the internalization of social media pressure. Furthermore, it suggested a concurrent parallel mediation effect involving mindfulness abilities in the relationship between COVID-19-related distress and body satisfaction. Based on previous research revealing a relationship between body image and the following of appearance-based content in adolescents [27, 29, 30, 44], we focused on participants’ use of two video and image-sharing platforms with a lot of content related to appearance and widely used by youth, namely Instagram and TikTok. We will refer to them as appearance-focused social media, an umbrella term recently used by Maes and Vanderbosch to combine both platforms [44].

## Methods

### Participants and procedures

The sample consisted of 493 Canadian adolescents and young adults aged 15 to 18 years (mean = 16.7; *SD* = 0.60). Most participants identified with the Québécois culture (75.5%), while 29.6 identified as Canadian, 3.6% as East and West European, 3.2 as Latin/South American, 2.6% as Caribbean, 1.4% as Middle Eastern 1.0% as American, 1.0% as African, 1.0% as Asian, 0.4% as Indigenous, or others (3.8%). According to gender identity, 47.5% (*n* = 234) identified as girls, 50.9% (*n* = 251) as boys, and 1.6% (*n* = 8) as non-binaries, gender-fluid, two-spirit or other.

Data were collected as part of a larger ongoing study about sports participation and resilience in Canadian adolescents. For the present study, data obtained during the most recent wave of recruitment were used (between May 10th and June 14th, 2022). Recruitment was conducted in five different schools from urban, semi-urban and rural areas, and various socioeconomical backgrounds, to ensure sample diversity. During this study, participants completed a self-report and anonymous battery of questionnaires on the Qualtrics survey platform. Participants completed the questionnaires on electronic tablets provided by research assistants, during school hours. The average time to complete the whole questionnaire battery was 40 min, which included three attention-testing questions randomly scattered throughout the battery. Study participants were compensated with a reusable water bottle and entry into a raffle (50$ gift certificates). Before completing the questionnaires, participants received detailed information about the study and provided informed consent. In Quebec, adolescents aged 14 and older have the right to give their own consent [45]. The procedure was adapted to the COVID-19 safety measures in effect at the time (e.g., a medical form completed by research assistants before each recruitment day; the use of face masks during data collection, disinfection of the electronic tablets after every use). This research protocol was approved by the University of Quebec at Chicoutimi Research Ethics Committee and performed according to the principles of the Declaration of Helsinki.

Initially, *n* = 505 adolescents agreed to participate in the study. After exploring the data, participants were excluded because they failed all three attention-testing questions (*n* = 6) or responded very inconsistently suggesting a random and thoughtless completion (*n* = 1). Then, five participants were excluded because they were older than 18 years old, resulting in a final sample of 493 participants.

### Measures

The model’s components were assessed using diverse measures chosen based on their psychometric properties, availability in French, suitability for adolescents, and brevity, considering a reduced number of items aligned with the context of recruitment.

#### Sociodemographic Information

Participants provided demographic information, including their age, biological sex (male or female) and gender identity (boy, girl, non-binary, gender-fluid, two-spirit), ethnic background, and primary language.

#### COVID-19-Related distress

The COVID-19 Peritraumatic Distress Index (CPDI) [46] was used to assess the frequency of distress manifestations like anxiety, depression, specific phobias, cognitive changes, avoidance and compulsive behaviors, physical symptoms and decrease in social functioning, in response to the COVID-19 pandemic. In the present study, a shorter 8-item validated version was used to ease the completion [47](see Supplementary file 1). This 8-item version was answered on a 5-point Likert scale ranging from 0 (*Never*) to 4 (*Always*). A higher score indicated a greater distress in response to the COVID-19 pandemic. The measure demonstrated an adequate consistency, with a Cronbach’s alpha of 0.77.

#### Use of Appearance-focused social media

The use of appearance-focused social media was measured by asking participants how often they used Instagram and TikTok, separately. Responses were given on a 5-point Likert scale ranging from 1 (*Never*) to 5 (*Always*). Items were then combined to create a global score; in our study, the two items correlated significantly and positively, with a moderate magnitude (*r* = 0.40). This measure was previously developed by Rodgers, Slater and their colleagues [26], including a variety of social media platforms. As in their study among adolescents, this score was positively and significantly associated with an average self-reported amount of time spent on social media (in minutes) on a typical day (*r* = 0.38).

#### Internalization of Social Media pressure

Pressure from social media was assessed by using four items of the Pressure subscale of the Sociocultural Attitudes Towards Appearance Questionnaire-3 (SATAQ-3) [48] adapted to social media, as proposed by Rodgers and colleagues [26] (see Supplementary file 2). Participants were asked if they had felt pressure from social media to (1) lose weight, (2) exercise, (3) change their appearance, and (4) have a perfect body. Items were scored on a 5-point Likert-scale ranging from 1 (*definitely disagree*) to 5 (*definitely agree*). A total score was created by summing each score; a higher score indicates a higher level of pressure from social media. In the present study, the scale showed an excellent internal consistency, with a Cronbach’s alpha of 0.93.

#### Body satisfaction

Body satisfaction was assessed using three items from the Body Change Inventory [49]. Participants were asked to indicate to what extent they were satisfied with their (1) weight, (2) shape and (3) muscle size using a 5-point Likert-scale ranging from 0 (*very unsatisfied*) to 4 *(very satisfied)*. Then, a total score of body satisfaction was created by summing the three items; a higher score indicates a greater body satisfaction. In the present study, the scale showed a good internal consistency, with Cronbach’s alpha of 0.85.

#### Mindfulness

Mindfulness was assessed with the brief version of the Child and Adolescent Mindfulness Measure (CAMM) [50]. This scale measures dispositional mindfulness, which represents the awareness of thoughts and feelings in the present moment. The 5-item brief version of the scale was previously validated in adolescents and demonstrated good psychometric properties. Participants were asked to answer on a 5-point Likert scale, ranging from 0 (*never true*) to 4 (*always true*). Scores were inverted and summed to create a total score of dispositional mindfulness. A higher score indicates a higher disposition for mindful abilities on a day-to-day basis. In the present study, the questionnaire showed an excellent internal consistency, with a Cronbach’s alpha of 0.90.

#### Disordered eating behaviors

Disordered eating behaviors, specifically restraint eating and binge eating, were assessed with items from the Eating Disorder Examination Questionnaire 6.0 (EDE-Q) [51] (see Supplementary file 3). In the present study, three items from the restraint subscale were used to assess restraint eating, and a mean score was then created (e.g., “How many of the past 28 days have you been deliberately trying to limit the amount of food you eat to influence your shape of weight (whether or not you have succeeded)?”). This scale showed good internal consistency, with Cronbach’s alpha of 0.89. Binge eating episodes were assessed using the following item on overeating and loss of control over eating during the last 28 days, “Over the past 28 days, on how many days have such episodes of overeating occurred (i.e., you have eaten an unusually large amount of food and a sense of loss of control at the time)?”. The total score ranged between 0 and 28.

### Statistical analyses

Descriptive statistics and correlational analyses were performed using the SPSS software version 28 [52]. Missing data at the item level was rare, and mean scores rounding was used where possible to reduce their impact. Multivariate outliers were subsequently detected and removed using the Mahalanobis distance and Cook’s distance values (*n* = 10).

The hypothesized model (presented in Fig. 1) was tested using path analyses in MPlus (version 8) [53]. We first tested a model suggesting paths between COVID-19-related distress, body satisfaction and disordered eating behaviors (restraint eating and binge eating), adding a parallel mediational effect of mindfulness, with appearance-focused social media use and social media pressure in series, in the relationship between COVID-19-related distress and body satisfaction. According to the mediational effects, the 95% confidence intervals (CI) for indirect effects were computed using 10,000 bootstraps resampling; if the calculated CIs included the zero, it meant that the proposed mediational effect was not statistically significant [54–56]. This set of analysis was tested using the maximum likelihood (ML) estimator, and the full information maximum likelihood (FIML) estimation method to deal with missing data [53]. Model fit was assessed using various fit indices, including the chi-square (χ^2^) test of model fit, root mean square error of approximation (RMSEA), standardized root mean square residual (SRMR), comparative fit index (CFI), and Tucker-Lewis index (TLI). Indices were assessed using the following guidelines: χ^2^, *p* >.05; SRMR < 0.08; RMSEA < 0.06; CFI and TLI > 0.90 [57, 58]. A multi-group gender-invariance analysis was then conducted using a chi-square difference test, between a constrained and an unconstrained model. A non-significant chi-squared difference suggested a model invariance between boys and girls. Thus, model testing was conducted for the overall sample, without considering the effect of gender. Furthermore, as age did not exhibit significant correlations with any variables of interest, we did not include it as a control variable to maintain a more parsimonious model.

## Results

Descriptive statistics and correlation analyses for variable of interest are presented in Table 1.

**Table 1:** Descriptive statistics and correlations for study variables

Outcomes	M	SD	1	2	3	4	5	6	7
1. COVID-19-related distress	6.87	5.19	-						
2. Use of appearance-focused social media	7.19	2.20	0.12**	-					
3. Internalization of social media pressure	8.64	5.07	0.40**	0.27**	-				
4. Mindfulness	12.44	5.33	− 0.47**	− 0.05	− 0.46**	-			
5. Body satisfaction	7.44	2.80	− 0.32**	− 0.11*	− 0.43**	0.42**	-		
6. Dietary restraint	0.85	1.51	0.17**	0.11*	0.38**	− 0.26**	− 0.34**	-	
7. Binge eating episodes	1.48	3.85	0.24**	0.10*	0.29**	− 0.25**	− 0.35**	0.28**	-

*Note.* *N* = 493 (234 girls; 251 boys)

*p <.05. **p <.01

### Path analyses

**Fig. 2:**
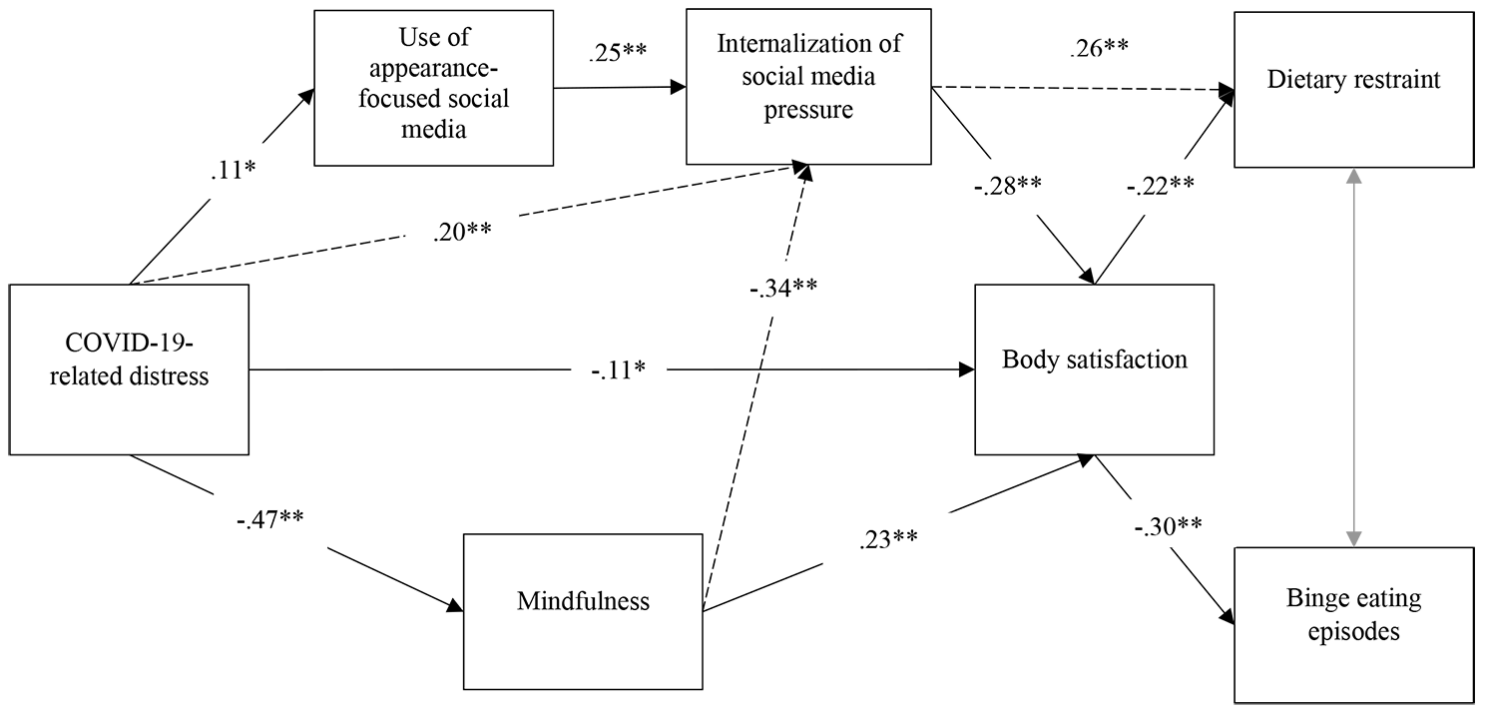
Modified Model

While all paths were found to be significant (with the exception of the correlation between dietary restraint and binge eating episodes), the initial model, illustrated in Fig. 1, revealed a poor fit to the data: χ2 (12) = 167.91, *p* <.001, SRMS = 0.12, RMSEA = 0.17, CFI = 0.71, TLI = 0.50. We explored modification indices using maximum likelihood with robust standard errors (MLR) to improve the model fit. We gave a theoretical consideration to the proposed paths and modified the initial model accordingly. Moreover, we ensured that the added pathways align with the model’s directionality (see Fig. 2). First, the non-significant path between dietary restraint and binge eating episodes was removed (*r* =.09) (pale arrow). Then, we added two paths towards internalization of social media pressure, one starting from COVID-19-related distress and the other from mindfulness (dashed arrows). These additions revealed a total of four significant indirect effects on the relationship between COVID-19-related distress and body satisfaction: (1) internalization of social media pressure (standardized estimate = − 0.06, 95% C.I. [− 0.09, − 0.03]); (2) mindfulness (standardized estimate = − 0.11, 95% C.I. [− 0.16, − 0.06]); (3) appearance-focused social media use and internalization of social media pressure (standardized estimate = − 0.01, 95% C.I. [− 0.02, − 0.001]); (4) mindfulness and internalization of social media pressure (standardized estimate = − 0.04, 95% C.I. [− 0.07, − 0.03]). In addition, the direct path from COVID-19-related distress to body satisfaction remained significant (standardized estimate = − 0.11, 95% C.I. [− 0.21, − 0.01]). Finally, we also added a path between internalization of social media pressure and dietary restraint (dashed arrow). The final model revealed a good fit to the data with excellent indices, *χ*^2^ (10) = 15.39, *p* =.12, SRMS = 0.03, RMSEA = 0.03, CFI = 0.99, TLI = 0.98.

## Discussion

The present study aimed to deepen our understanding of the relationships between the COVID-19-related distress, body image and disordered eating behaviors among adolescents. To our knowledge, this study is the first to elaborate and test a model in which COVID-19-related distress predicted body satisfaction, which in turn predicted disordered eating behaviors (dietary restraint and binge-eating episodes). Globally, our findings support the relationships among these variables and highlight the mediational roles of appearance-focused social media use, internalization of social media pressure, and mindfulness, in the relationship between COVID-19-related distress and body satisfaction. However, the model we originally hypothesized displayed a poor fit to the data. In the following paragraphs, we initially describe the paths from the initial model that persisted into the final model, and then we delved into the adjustments made to achieve a well-fitted model.

In the first part of the model, the relationship between COVID-19-related distress and lower body satisfaction was simultaneously mediated by lower mindfulness on one hand, and by an increased use of appearance-focused social media and greater internalization of social media pressure in series on the other. Those results align with our initial assumptions about the potential factors underlying the association between COVID-related distress and body satisfaction. In the second part of the model, regression estimates were significant and negative, suggesting that a lower body satisfaction predicted more disordered eating behaviors. Overall, results of the present study are consistent with previous findings showing that more COVID-19-related distress was significantly associated with a lower body satisfaction and more disordered eating behaviors [18], greater use of appearance-focused social media and internalization of social media pressure [27, 28], and lower mindfulness [59, 60]. Regarding social media, the serial mediation implied that the frequency of appearance-focused social media use, including Instagram and TikTok, significantly explained the relationship between COVID-19-related distress and the internalization of social media pressure. This finding supports the importance of media exposure to explain the impact of the COVID-19 pandemic on body image and eating behaviors, as proposed in the theoretical pathways elaborated by Rodgers, Lombardo and their colleagues [21]. Specifically, our findings enhance this model, suggesting that mindfulness might be a key process explaining the link between COVID-19-related distress and body image and eating behaviors. This inclusion highlights a potential intervention target that might prevent or alleviate this body dissatisfaction and disordered eating. However, longitudinal designs are necessary to confirm the directionality and temporality of these relationships.

In addition to these significant pathways, in the final model we introduced three new paths and removed one from the originally hypothesized model to improve the model’s fit. The most important modification was the addition of two paths in the first part of the model, that is, from COVID-19-related distress and mindfulness to internalization of social media pressure. The incorporation of these pathways emphasizes the oversimplification of the relationships between variables in our initial model. For instance, it illustrates the intricate nature that underscores the potential impact of the COVID-19-related distress and mindfulness on the internalization of social media pressure. While the parallel mediational effects might offer a more streamlined model, they may not authentically capture the complexity of these phenomena in real-life situations. More specifically, this addition led to the further identification of two significant indirect paths in those already present between COVID-19-related distress and body satisfaction. The first of these indirect paths suggest that although COVID-19-related distress was associated with the internalization of social media, through the use of appearance-focused social media, it was also directly associated with this internalization, which in turn was associated with greater body dissatisfaction. The second indicates that COVID-19-related distress was associated with body satisfaction through mindfulness and the internalization of social media pressure in series. This mediational effect implies that the relationship between COVID-19-related distress and the internalization of social media pressure was significantly explained by mindfulness. Thus, it is possible to infer that the experience of body dissatisfaction in reaction to the COVID-19-related distress in adolescents could be explained by the combined effect of a lower mindfulness and a greater tendency to internalize social media pressure. Overall, all indirect paths in the first part of the model suggest that in reaction to a greater distress from the pandemic, adolescents who frequently use social media exposing appearance-related content, and who have lower mindfulness abilities, could be more prone to internalize the pressure about body image and ultimately be less satisfied with their own appearance. Taken together, our results highlight the relationship between the internalization of social media pressure and body satisfaction, and the potential contribution of mindfulness and appearance-focused social media use in this internalization. Indeed, they also revealed that the use of appearance-focused social media alone is a weak predictor of body satisfaction and must be accompanied by an internalization of the pressure they convey about body image, which is consistent with previous explanatory models on body dissatisfaction and disorders eating behaviors in adolescents [61, 62]. These findings are also in line with a recent study that highlighted the importance of social media content in opposition to duration/frequency. Specifically, Sanzari and colleagues [29] concluded that the duration of social media use was not a significant predictor of negative outcomes; individuals exposed to more content about weight loss, however, reported more body image concerns and disordered eating behaviors. It is also possible that our self-report and brief measure of social media use influenced this result, and that a more objective measure would have revealed a direct pathway from social media use to body satisfaction.

In addition, to improve the final model, we removed the non-significant correlation between both eating behaviors (dietary restraint and binge eating episodes). The absence of a significant relationship within the model suggests that body satisfaction correlated independently with these disordered eating behaviors when analyzed alongside with COVID-19-related distress, social media variables, and mindfulness. Notably, at the bivariate level, a significant correlation between dietary restraint and binge eating episodes (*r* =.28, *p* < 0.01) was observed. Consequently, it is plausible that dietary restraint and binge eating represent distinct responses to body image concerns and associated emotions when linked to COVID-19-related distress, and perhaps could be associated with different behavioral profiles (such as controlled versus dysregulated) [63]. This finding requires further validation to confirm whether distinct trajectories of eating behaviors in response to a major stressor can be identified in connection with body dissatisfaction. It is essential to examine whether this outcome is specific to our study or if it remains consistent across different contexts. Finally, a path was added directly between internalization of social media pressure and dietary restraint, which is consistent with a previous model with similar construct among adolescents [26]. It emphasizes the direct relationship between the internalization of social media pressure and dietary restraint, supporting the significant impact of this pressure on adolescents’ eating behaviors, and potentially driven by factors other than body dissatisfaction. Given the cross-sectional nature of our study, this refined model requires further validation through longitudinal designs to solidify these pathways, indirect effects, and their directionality.

This study has several limitations, including its cross-sectional design, which prevented the examination of causal relationships between our variables and drawing conclusions about their directionality. Even though participant retention was complicated during the COVID-19 pandemic, we hope that researchers will reproduce our model using a longitudinal design. Another important limitation was the use of self-reported measures only. Although this type of measure had many benefits during the pandemic and allowed for valuable data to be collected, their subjective nature and inherent biases may have influenced our results and conclusions (e.g., social desirability, stigma, selective recall, comprehension difficulties). It would be interesting to assess the use of appearance-focused social media and disordered eating behaviors with more detailed and sensitive measures (e.g., content analysis for social media), including semi-structured interviews and/or ecological momentary assessments (EMA). Another limitation pertains to the utilization of abbreviated measures, subscales, or individual items. While advantageous for this study, this approach may have restricted the depth of our conclusions. Specifically concerning eating behaviors, it hindered our ability to distinguish between adolescents with and without eating disorders, as well as other behaviors associated with eating disorders (such as excessive exercising, purging, etc.). Finally, we did not include potential moderators that could have specified the model, such as socioeconomic status and family environment, that certainly played a role in the way adolescents experienced the pandemic.

## Conclusions and clinical implications

The present findings highlight the complexity of the relationships between the COVID-19-related distress, body satisfaction and disordered eating behaviors. The processes suggested by the final model revealed the potential core contribution of internalization of social media pressure and mindfulness abilities. Specifically, in reaction to a greater distress related to the COVID-19 pandemic, lower mindfulness abilities seem to contribute to the process of internalizing social media pressure, to body dissatisfaction and ultimately to disordered eating behaviors. Thus, instead of only focusing on the time adolescents spend on social media, it could be more effective to assess and develop interventions on the way they watch or receive it. For example, in a context of high emotional distress, a teenager could only need one picture or video to be triggered and to enter in a body image concerns-disordered eating behaviors loop, without necessarily spending many hours on it. In clinical contexts, it supports the use of various therapeutic approaches including mindfulness abilities (e.g., Dialectical Behavior Therapy, Acceptance and Commitment Therapy, Mindfulness-Based Cognitive Therapy). Accordingly, an interesting avenue would be to promote intervention programs using a mindfulness-based approach in the context of social media use specifically, to help youth use and react mindfully to social media content (e.g., Mindful Connections) [64]. We could also expand these programs for educational or preventive purposes to empower youth and minimize the potential impacts of media social use on body image and eating behaviors. It represents an interesting avenue to promote psychological adaptation and resiliency during stressful times, and to prevent the development of mental health issues.

Even though the end of this pandemic seems in sight, we think that this model is relevant for the post-pandemic era, during which we expect an increase in psychological difficulties and demands for specialized services [65], including body image concerns and disordered eating behaviors. Indeed, it supports the need for mental health workers to assess the use of appearance-focused social media and the internalization of social media pressure, and mindfulness abilities, especially among adolescents who present with body image and eating behavior disturbances. This model can also inform us on how some adolescents may react to stressful situations, and mechanisms possibly leading to experiencing body image concerns and eating behavior disturbances.

### Electronic supplementary material

Below is the link to the electronic supplementary material.

Supplementary material 1

## Data Availability

The data are not publicly available because of ethical restrictions, they were used under license for the present study. The data presented in this study are available upon request.
