# Supplementary material for: COVID-19-Related distress, body image, and eating behaviors: a cross-sectional explanatory model
Source: BMC Psychol. 2024 Mar 2;12:117. doi: 10.1186/s40359-024-01613-z (PMC10908208; doi:10.1186/s40359-024-01613-z)
Supplement: Supplementary file 1 — Supplementary material 1 [file 40359_2024_1613_MOESM1_ESM.docx]

**Supplementary files**

**Supplementary file 1:** *COVID-19 Peritraumatic Distress Index* (CPDI) (Short version)

**During the COVID-19 period**, for each of the following statements, select the answer that most applies to you in the week.

|  | Never | Rarely | Sometimes | Often | Most of the time |
| --- | --- | --- | --- | --- | --- |
| 1. I can’t stop imagining myself or my family becoming infected and the idea scares me. |  |  |  |  |  |
| 1. I feel sad for people who have COVID-19 and their families. |  |  |  |  |  |
| 1. I feel angry about people around me, governors, or media. |  |  |  |  |  |
| 1. I am losing faith in the people around me. |  |  |  |  |  |
| 1. I tend to believe the negative news about COVID-19, but be skeptical about the good news. |  |  |  |  |  |
| 1. I mostly share the negative news about COVID-19. |  |  |  |  |  |
| 1. I feel sluggish. |  |  |  |  |  |
| 1. I find it hard to concentrate. |  |  |  |  |  |

Supplementary file 1 includes the shorter 8-item version of the CPDI used in the present study.

**Supplementary file 2:** *Pressure subscale of the Sociocultural Attitudes Towards Appearance Questionnaire-3* (SATAQ-3) (Adapted to social media)

| **I feel pressure from the social media to…** | Definitely disagree | Agree | Uncertain | Disagree | Definitely agree |
| --- | --- | --- | --- | --- | --- |
| 1. Lose weight |  |  |  |  |  |
| 1. Exercise |  |  |  |  |  |
| 1. Improve my appearance |  |  |  |  |  |
| 1. Have a perfect body |  |  |  |  |  |

Supplementary file 2 includes the 4-item SATAQ-3 adapted to social media used in the present study.

**Supplementary file 3:** *Eating Disorder Examination – Questionnaire (EDE-Q)* (Short version)

**The following questions are concerned with the past four weeks (28 days) only. Please read each question carefully.**

| **On how many of the past 28 days…** | No days | 1-5 days | 6-12 days | 13-15 days | 16-22 days | 23-27 days | Everyday |
| --- | --- | --- | --- | --- | --- | --- | --- |
| 1. Have you been deliberately trying to limit the amount of food you eat to influence your shape or weight (whether or not you have succeeded)? |  |  |  |  |  |  |  |
| 1. Have you tried to exclude from you diet any foods that you like in order to influence your shape and weight (whether or not you have succeeded)? |  |  |  |  |  |  |  |
| 1. Have you tried to follow definite rules regarding your eating (for example, a calorie limit) in order to influence your shape or weight (whether or not you have succeeded)? |  |  |  |  |  |  |  |

**Please fill in the appropriate number in the box on the right. Remember that the questions only refer to the past four weeks (28 days).**

**Over the past four weeks (28 days)…**

1. Over the past 28 days, how many times have you eaten what other people would regard as an unusual large amount of food (given the circumstances)? …………………………..
2. On how many days did you have the sense of having lost control over your eating (at the time that you were eating)? …………………………...
3. Over the past 28 days, on how many **DAYS** have such episodes of overeating occurred (i.e., you have eaten an unusually large amount of food and have had a sense of loss of control at the time)? …………………………..

Supplementary file 3 includes six items from the EDE-Q, the first three for assessing dietary restraint and the other three to assess binge eating episodes.
